# 710 Specific Patterns of Vital Sign Fluctuations Predict Bloodstream Infection in Pediatric Burn Patients

**DOI:** 10.1093/jbcr/irac012.264

**Published:** 2022-03-23

**Authors:** Farzin Sadeq, Jonah Poster, Joan M Weber, Maggie D Begis, Robert L Sheridan, Korkut Uygun

**Affiliations:** Shriners Hospitals for Children Boston, Boston, Massachusetts; Shriners Hospitals for Children Boston, Newton, Massachusetts; Shriners Hospitals for Children Boston, Boston, Massachusetts; University of New Hampshire, Amesbury, Massachusetts; Shriners Hospitals for Children Boston, Boston, Massachusetts; Massachusetts General Hospital, Boston, Massachusetts

## Abstract

**Introduction:**

Early recognition of the clinical signs of bloodstream infection in pediatric burn patients is key to improving survival rates in the burn unit. The objective of this study was to propose a simple scoring criteria that used readily available temperature, heart rate (HR) and mean arterial pressure (MAP) data to accurately predict bloodstream infection in pediatric burn patients.

**Methods:**

A retrospective chart review included 100 patients admitted to the pediatric burn unit for >20% total body surface area (TBSA) burn injuries. Each patient had multiple blood culture tests, and each test was treated as a separate and independent “infection event” for analysis. The time at each blood culture draw was time 0 for that event, and temperature, HR and MAP data was collected for 24 hours after the blood culture was drawn. “Infection events” included in this study had at least six complete sets of temperature, HR and MAP data entries. Median temperature, HR and MAP, as well as mean fever spikes, HR spikes and MAP dips, were compared between infection group (positive blood cultures) and control group (negative blood cultures). These vital sign fluctuations were evaluated individually and as a combination of all three as timely predictors of bloodstream infection. In addition, we tested the prediction of Gram-negative bacteria versus Gram-positive or fungi present in blood cultures.

**Results:**

Patients in the infection group had significantly higher median temperatures (*p*< 0.001), mean fever spikes (*p*< 0.001) and mean HR spikes (*p*< 0.001), compared to the control group. Using the combination scoring criteria to predict bloodstream infection, the strongest predictive values in the 24-hour timeframe had high sensitivity (93%) and specificity (81%). The predictive test metric based on vital sign spikes predicted Gram-negative bacteria, but with limited sensitivity (57%) and specificity (44%).

**Conclusions:**

This study found that using a combination scoring criteria of fever spikes, HR spikes and MAP dips predicted bloodstream infection in pediatric patients with burn injuries with 87% accuracy, which may justify its use in resource-poor environments, or in cases where practical supporting evidence is needed for preemptive antibiotic treatment before culture results are available.

